# Supplementary material for: Caveolin 1 Regulates the Tight Junctions between Sertoli Cells and Promotes the Integrity of Blood–Testis Barrier in Yak via the FAK/ERK Signaling Pathway
Source: Animals (Basel). 2024 Jan 5;14(2):183. doi: 10.3390/ani14020183 (PMC10812639; doi:10.3390/ani14020183)

Figure S5. Figure 7H

FAK 129 KDa

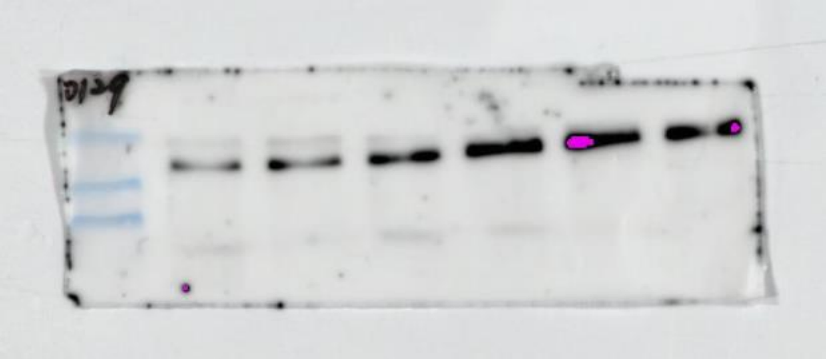

SRC   55 KDa

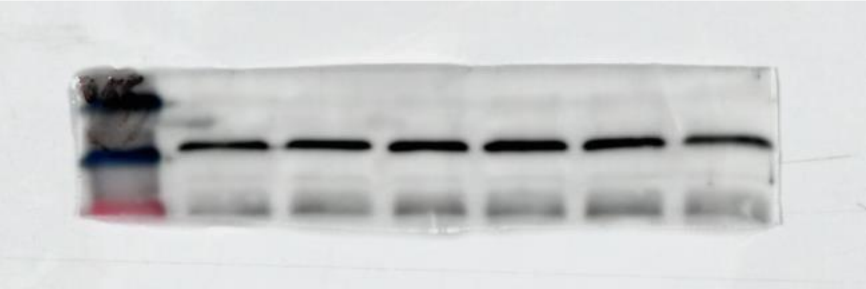

p-SRC   60 KDa

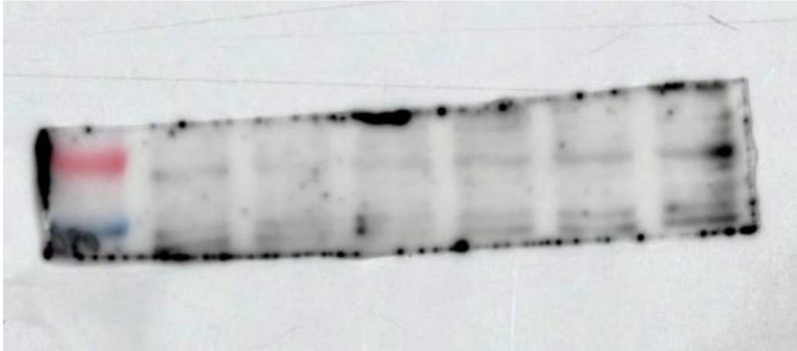

JNK123   45 KDa

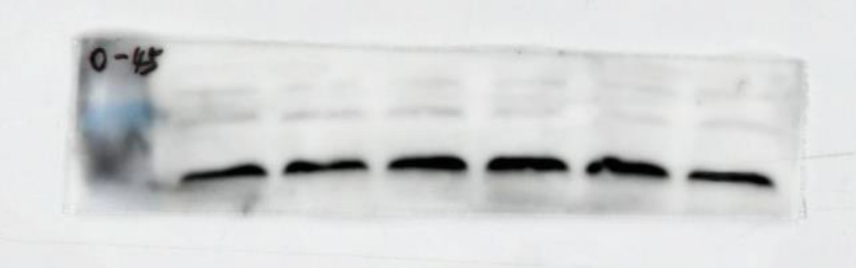

ERK1/2   41 KDa

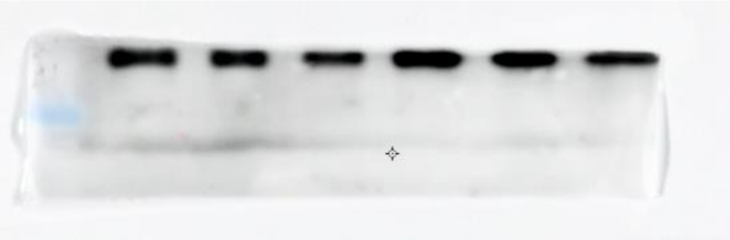

p-ERK1/2   41 KDa

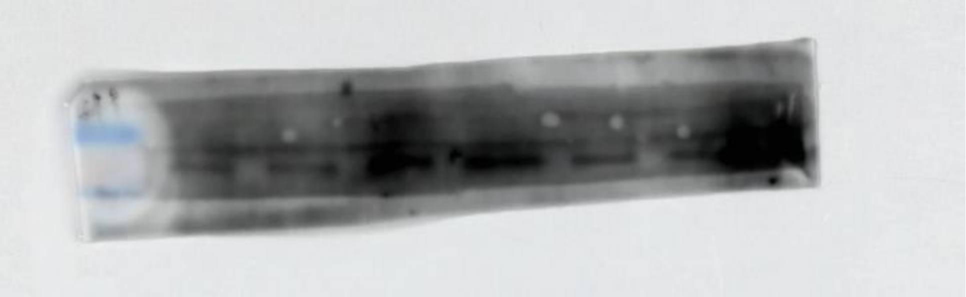

ZO-1   230 KDa

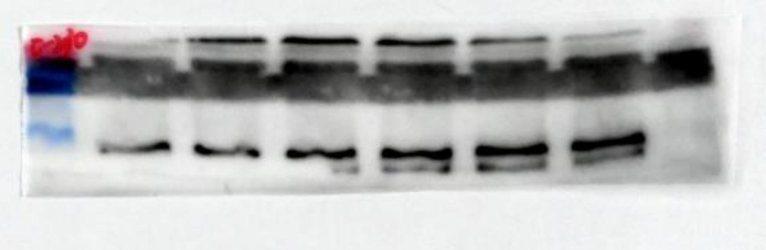

Occludin   59 KDa

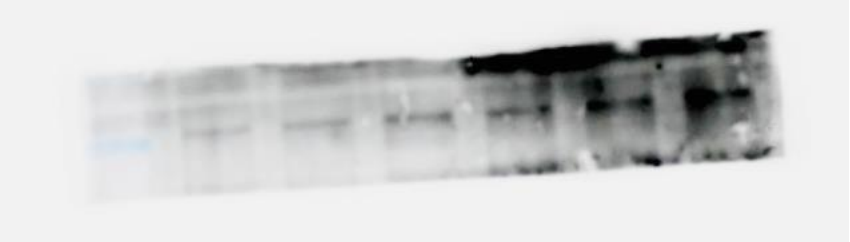

Claudin-11   22 KDa

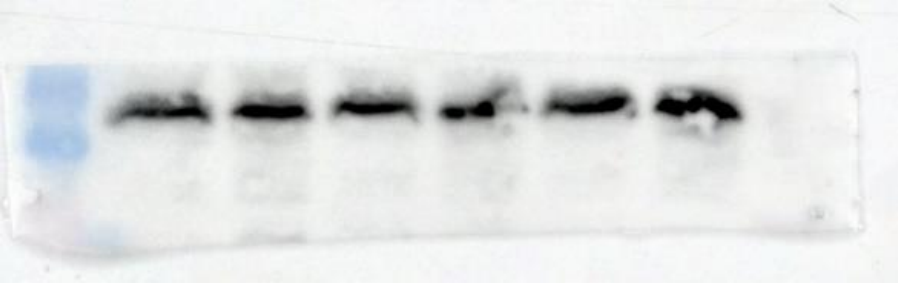

GAPDH   36 KDa

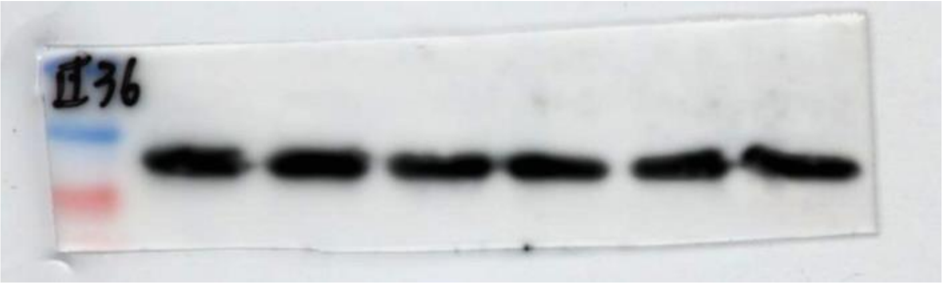

Supplement: Supplementary file 1 [file animals-14-00183-s001.zip › Figure S5.pdf]
