# Supplementary material for: Caveolin 1 Regulates the Tight Junctions between Sertoli Cells and Promotes the Integrity of Blood–Testis Barrier in Yak via the FAK/ERK Signaling Pathway
Source: Animals (Basel). 2024 Jan 5;14(2):183. doi: 10.3390/ani14020183 (PMC10812639; doi:10.3390/ani14020183)

Figure S6. Figure 8H

FAK 129 KDa

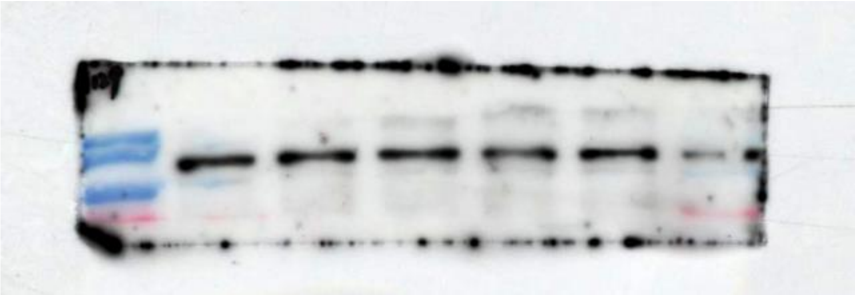

SRC 55 KDa

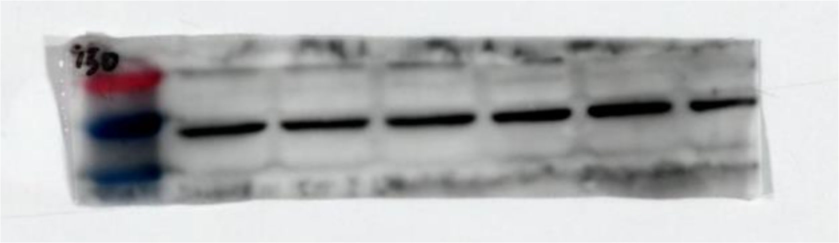

p-SRC 60 KDa

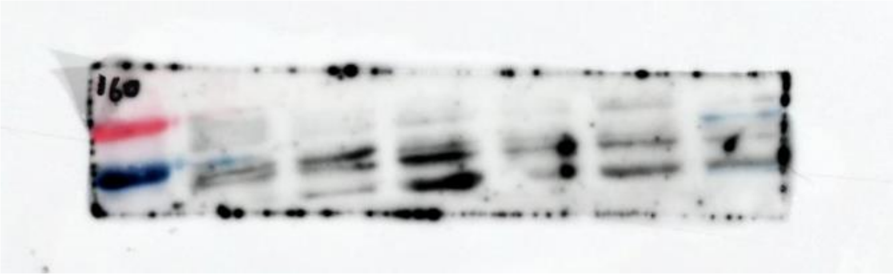

JNK123 45 KDa

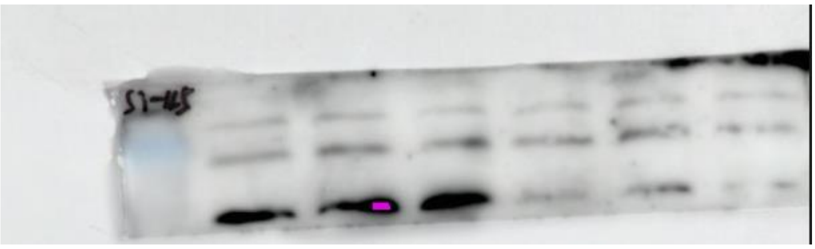

ERK1/2 41 KDa

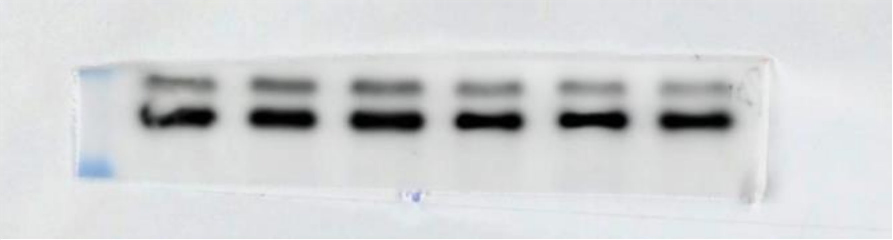

p-ERK1/2 41 KDa

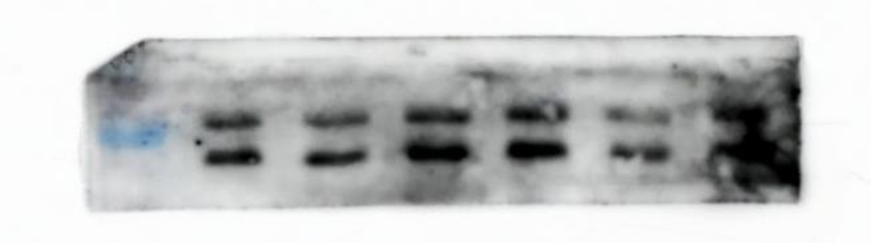

ZO-1 230 KDa

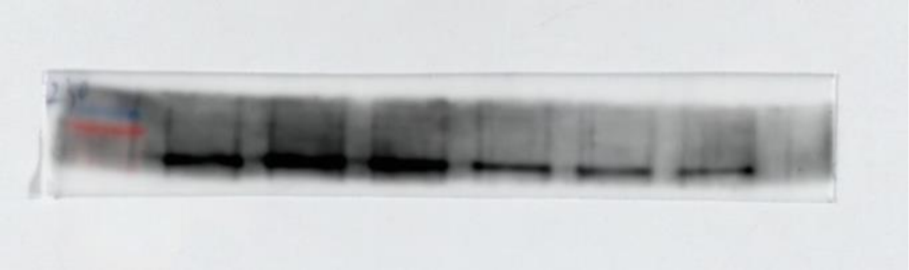

Occludin 59 KDa

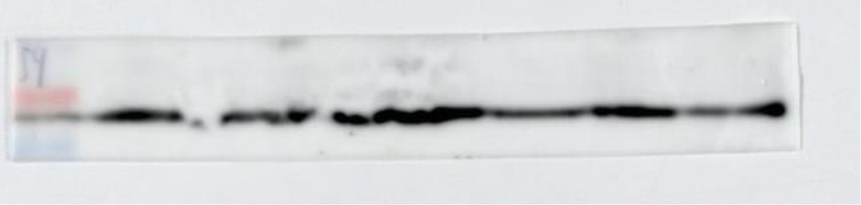

Claudin-11 22 KDa

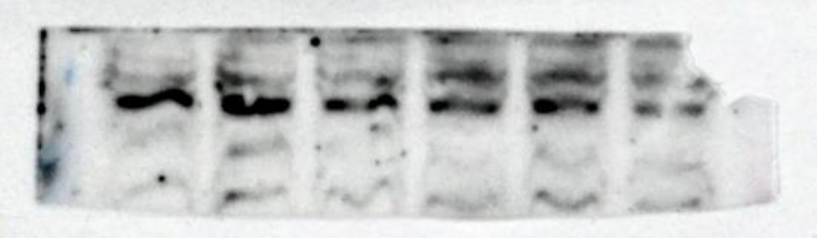

GAPDH 36 KDa

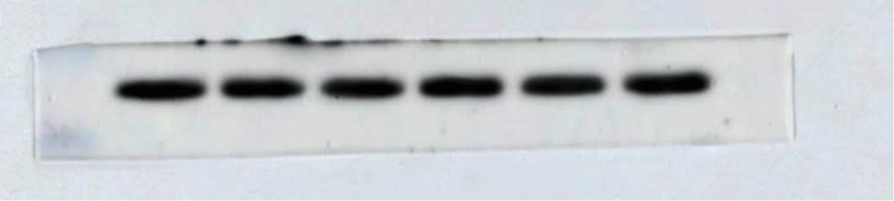

Supplement: Supplementary file 1 [file animals-14-00183-s001.zip › Figure S6.pdf]
