# Supplementary figures and images for: Caveolin 1 Regulates the Tight Junctions between Sertoli Cells and Promotes the Integrity of Blood–Testis Barrier in Yak via the FAK/ERK Signaling Pathway
Source: Animals (Basel). 2024 Jan 5;14(2):183. doi: 10.3390/ani14020183 (PMC10812639; doi:10.3390/ani14020183)

**Figure S1. Figure 1C**

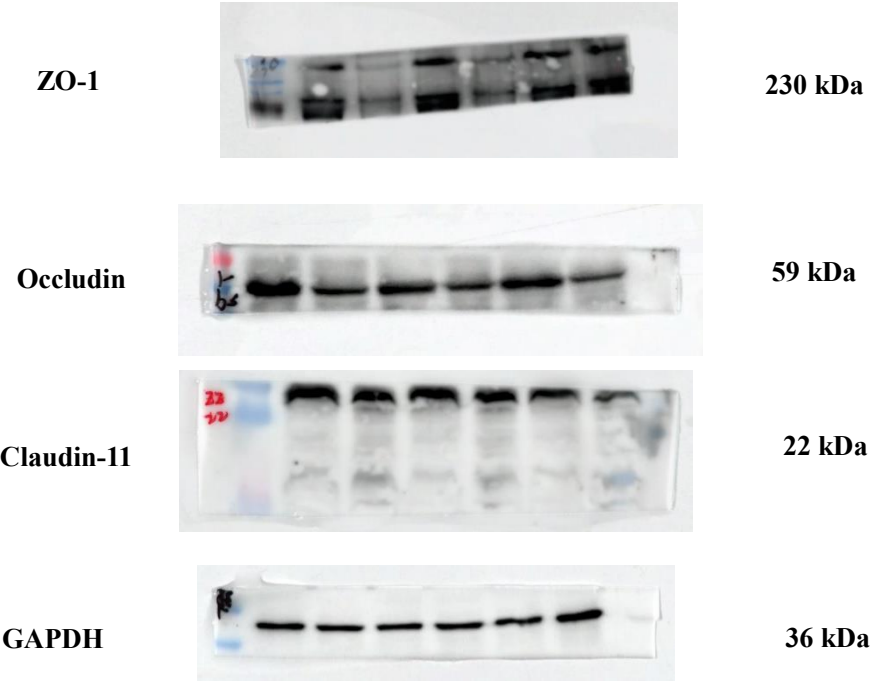

Supplement: Supplementary file 1 [file animals-14-00183-s001.zip › Figure S1.pdf]

**Figure S2. Figure 4C**

GAPDH 36 KDa

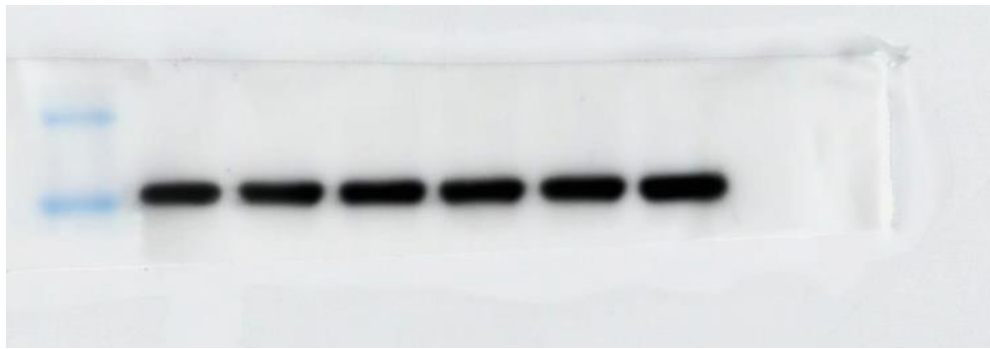

TGFB1 44 KDa

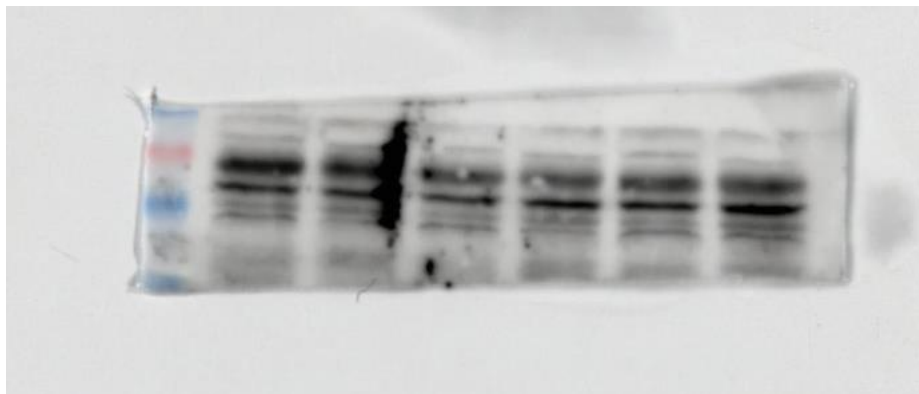

CAV1 22 KDa

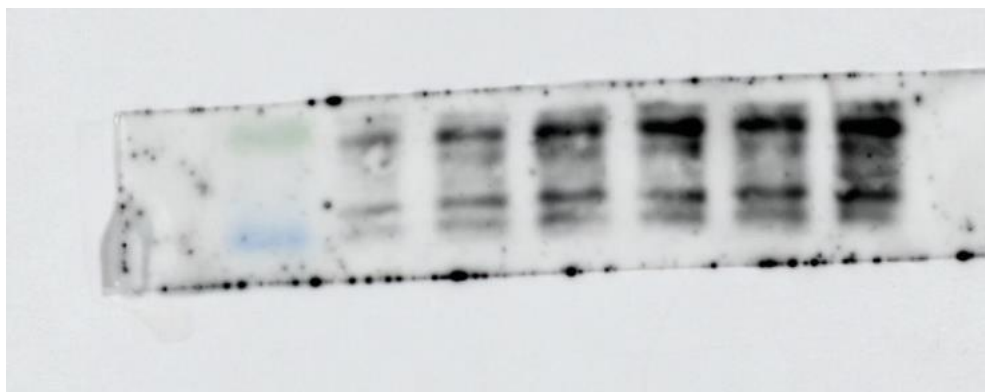

Supplement: Supplementary file 1 [file animals-14-00183-s001.zip › Figure S2.pdf]

**Figure S3. Figure 5D**

GAPDH 36 KDa

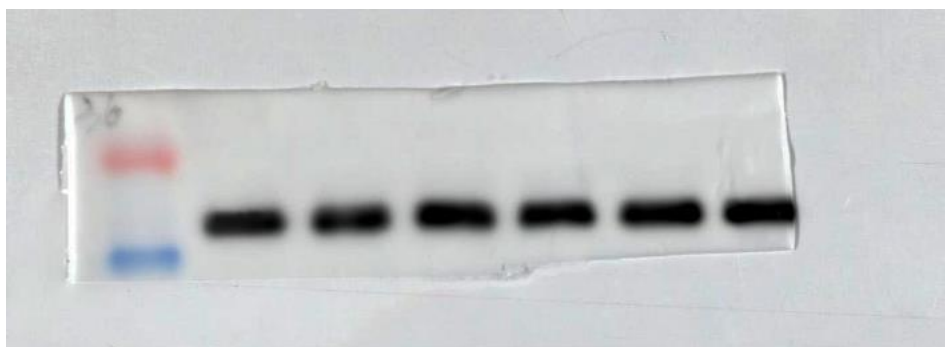

CAV1 22 KDa

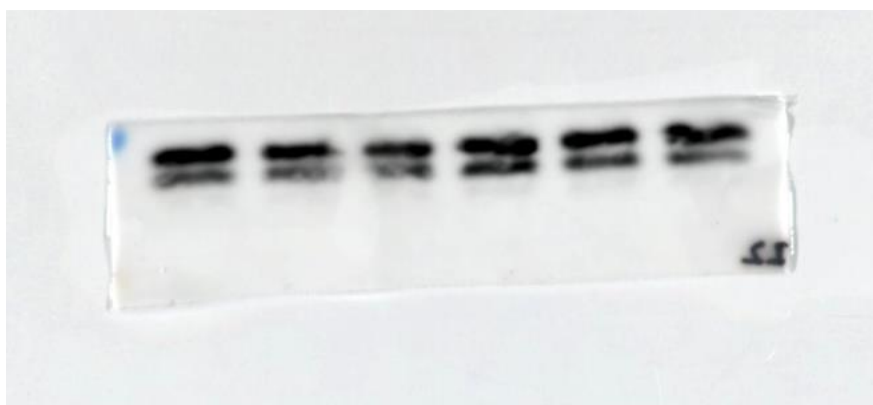

Supplement: Supplementary file 1 [file animals-14-00183-s001.zip › Figure S3.pdf]

**Figure S4. Figure 6B**

GAPDH-1 36 KDa

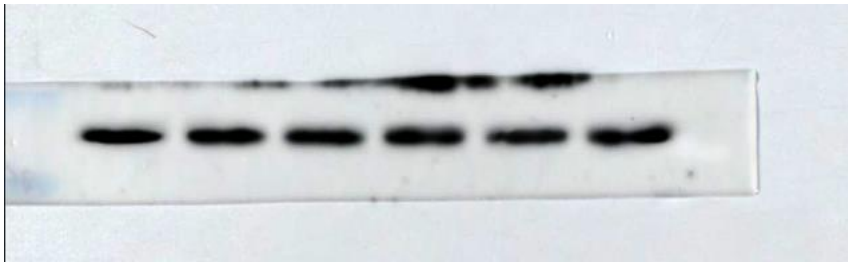

Si-CAV1-1 22 KDa

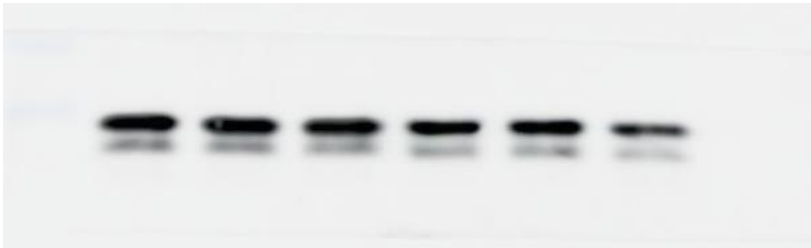

GAPDH-2 36 KDa

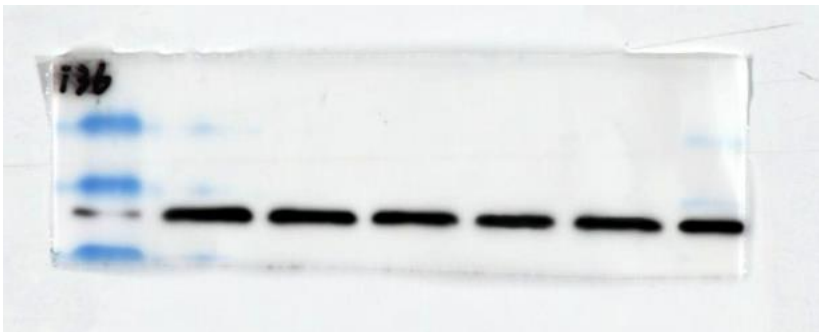

Si-CAV1-2 22 KDa

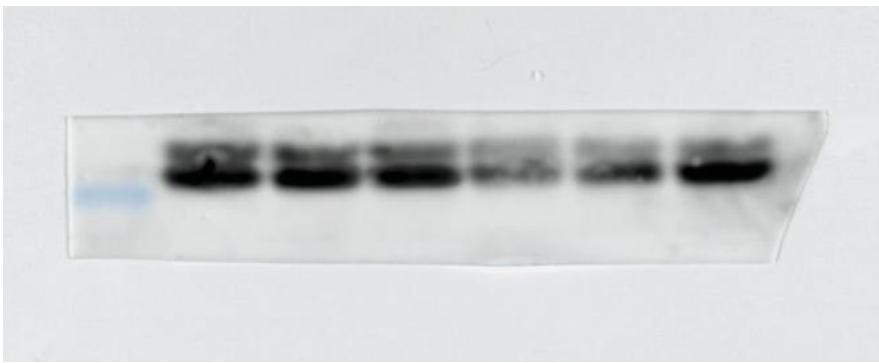

Supplement: Supplementary file 1 [file animals-14-00183-s001.zip › Figure S4.pdf]
